# mTOR Inhibitors in Cancer: What Can We Learn from Exceptional Responses?^☆^

**DOI:** 10.1016/j.ebiom.2014.12.011

**Published:** 2014-12-26

**Authors:** David J. Kwiatkowski, Nikhil Wagle

**Affiliations:** a Department of Medicine, Brigham and Women's Hospital, Boston, MA 02115, United States; b Dana Farber Cancer Institute, Boston, MA 02115, United States; c Broad Institute of MIT and Harvard, Cambridge, MA 02142, United States

Rapamycin is a macrolide antibiotic originally isolated from *Streptomyces hygroscopicus*, a bacterium isolated from a soil sample collected on Easter Island. It was found to suppress the immune system by blocking T-cell activation and proliferation, and was approved by the FDA for prophylaxis against organ rejection in renal transplant patients in 1999. Molecular studies first defined a role for rapamycin in complex with a small isomerase FKBP12 in inhibiting the activity of kinases TOR1 (TOR = Target of Rapamycin) and TOR2 in yeast. Further studies showed that rapamycin-FKBP12 binds to and inhibits the homologous kinase in mammalian cells, mTOR. More recently, it has been recognized that mammalian mTOR forms two large multi-subunit protein complexes, mTORC1 and mTORC2, that have independent non-overlapping phosphorylation targets, and that mTORC1 is the target of rapamycin-FKBP12 inhibition, while mTORC2 is not (Fig. 1).

The ability of rapamycin to inhibit phosphorylation and activation of the S6 kinases suggested early on that it might have cancer growth inhibitory properties. Pharmaceutical efforts led to the development of multiple related drugs, collectively termed rapalogs, including everolimus and temsirolimus. After Phases I–II studies showed promise, randomized clinical trials demonstrated the benefit of temsirolimus and everolimus for treatment of patients with metastatic renal cell carcinoma. Everolimus has since also been FDA-approved for neuroendocrine tumors of pancreatic origin (PNET), breast carcinoma, and subependymal giant cell astrocytomas (SEGA) associated with tuberous sclerosis.

A major role for the tumor suppressor genes, *TSC1* and *TSC2*, in the regulation of the S6Ks was first identified in 2002. Subsequent studies led to a current understanding that the TSC protein complex serves a critical role in the regulation of mTORC1 activity, through serving as a GTPase-activating protein (GAP) for RHEB (Fig. 1). Mutations in *TSC1* or *TSC2* cause the human genetic disease Tuberous Sclerosis Complex (TSC), and studies in both mouse models and human TSC-related tumors demonstrated that mTORC1 was highly activated in these tumors. After encouraging initial trials, rapamycin or everolimus have been shown to have benefit for several TSC tumors or related diseases, including renal angiomyolipoma, lymphangioleiomyomatosis, and subependymal giant cell astrocytoma in recent randomized clinical trials (Bissler et al., 2013, Mccormack et al., 2011).

Expanded clinical investigation of rapalogs in various cancers has led to a recognition that occasional patients display dramatic clinical responses. The first cancer type in which this was seen was PEComa, a rare sarcoma subtype in which mutations in *TSC1* or *TSC2* are common. Several PEComa patients have shown complete response (CR) to rapalogs lasting over a year including those with massive tumors (Wagner et al., 2010, Dickson et al., 2013). More recently, Solit and colleagues reported a sustained CR in a patient with metastatic bladder cancer that has now lasted over 4 years (Iyer et al., 2012). TSC1 inactivating mutations have been known in bladder cancer for many years, and this responding patient had a truncating mutation in TSC1. This discovery kicked off the current ‘exceptional responder’ initiative promoted by the NCI. Several other recent reports have identified patients with major responses to rapalog therapy, including another patient with bladder cancer shown to have two activating *MTOR* mutations and a patient with anaplastic thyroid cancer shown to have an inactivating mutation in *TSC2* (Wagle et al., 2014a, Wagle et al., 2014b). Furthermore a recent report of exceptional responders to rapalogs among patients with renal cell carcinoma identified inactivating *TSC1* and/or activating *MTOR* mutations in 3 of 5 patients (Voss et al., 2014).

In aggregate these studies have identified inactivating mutations in *TSC1* or *TSC2*, or activating mutations in *MTOR*, as correlating with and presumably accounting for the exquisite sensitivity to rapalogs that have been seen clinically. Notably, none of *PI3K*, *PTEN*, or *AKT1*/*AKT2*/*AKT3* mutation has been associated with response to date, likely reflecting their upstream position in this pathway, and their other effects in addition to mTORC1 activation (Fig. 1). Mutations in other components of this pathway, including inactivating mutations in *DEPDC5*, *NPRL2*, and *NPRL3*, and an activating mutation (Y35N) in *RHEB* have been shown to lead to strong mTORC1 activation in vitro, including analysis of cancer cell lines in some cases (Bar-Peled et al., 2013, Grabiner et al., 2014).

So can we predict who will respond to rapalog therapy? At present we cannot. However, we can make some observations and predictions, and formulate hypotheses for future studies. First, it is clear that there is a striking correlation between inactivating mutations in *TSC1* or *TSC2*, and activating mutations in *MTOR*, and response to rapalog therapy in several cancer types. Second, we predict that cancers with activating mutations in *RHEB*, or inactivating mutations in *DEPDC5*, *NPRL2*, *and NPRL3*, will show similar strong response to rapalogs. Third, it is already clear that not all cancers with mutations in members of this pathway will show such extraordinary responses (Iyer et al., 2012). Indeed, there is likely to be a range of responses to rapalogs even for tumors with activating mutations in the mTOR pathway. There are likely several explanations for this phenomenon, including as yet unidentified secondary modifier mutations, the tumor cell genetic and epigenetic states, as well as the possibility that some apparent mutations represent background noise or are subclonal, and did not contribute substantially to tumor development. Fourth, it also apparent that not all rapalog responders have mutations in components of this pathway (Voss et al., 2014). Apart from the mundane possibility that mutations were missed, there is the more important possibility that alternative mechanisms contribute to response, including epigenetic silencing events affecting one or more of the genes encoding proteins that inhibit mTORC1 activation.

Nevertheless, studies of extraordinary responses to rapalogs suggest that routine screening of cancer patients for alterations in the mTOR pathway may be helpful to identify a subset of patients who are much more likely to respond to mTOR-pathway targeted therapies than other patients. The collective prevalence of mTOR pathway mutations is appreciable among the common cancers (2.8% on average), including many cancers in which rapalogs are rarely used (e.g. lung adenocarcinoma, lung squamous cell carcinoma, melanoma, pancreatic adenocarcinoma, uterine carcinoma all have a prevalence > 1%) — highlighting the possibility of additional groups of patients who might benefit from these drugs. These observations have now spawned the so-called “basket" trials, clinical trials that enroll patients based on specific mutations rather than tumor types (e.g. NCT02201212, clinicaltrials.gov). Results of these trials should help to elucidate the factors that determine exquisite dependency on the mTOR pathway as well as the principles of extraordinary responses in general.

One challenge of rapalog basket trials is that the list of cancer genes and mutations that lead to sensitivity to mTOR inhibitors is incomplete and still evolving. Ideally, genomically-driven basket trials would have flexible entry criteria, permitting the range of genes and mutations to be dynamically modified during the trial, to take advantage of improved understanding as well as trial experience. In parallel, more detailed characterization of the functional effects of potential activating mutations in *MTOR* and *RHEB* (Grabiner et al., 2014) would be valuable in refining entry criteria for rapalog trials.

There is enormous diversity in the clinical response of patients to anti-cancer drugs and in most cases we do not understand why. Many agents in clinical trials “fail” and may be abandoned, yet, as with rapalogs, there are often a few patients in whom these agents have profound activity. Studies of exceptional responses demonstrate that genomic characterization of even a few patients with extraordinary responses can yield important insights. These studies could help us develop methods for matching patients to drugs, highlight effective uses for otherwise “failed” therapies, and design new therapeutic strategies. Findings from these studies may also help us understand mechanisms of therapeutic resistance when it emerges, and may help develop strategies to overcome such resistance (Wagle et al., 2014a). Unlike other large-scale cancer genomics efforts, identifying and characterizing the tumors from even a few extraordinary responses can lead to major insight and advances in cancer therapy.

## Figures and Tables

**Fig. 1:**
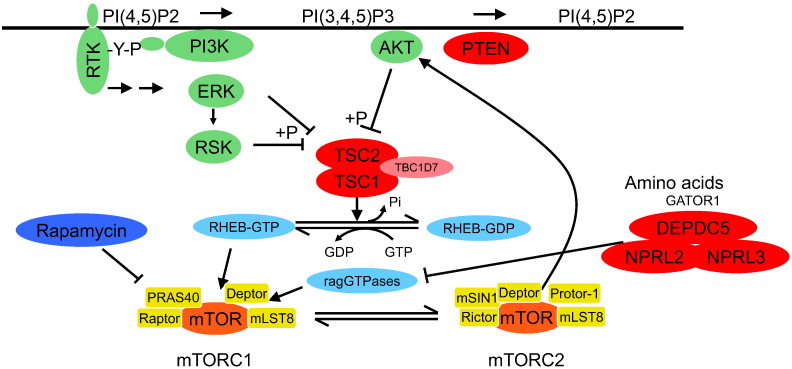
Regulation of mTORC1. The TSC complex, comprised of TSC1, TSC2, and TBC1D7 plays a critical role in regulating RHEB-GTP levels and thereby mTORC1 activity. The TSC complex is negatively regulated by phosphorylation by AKT, ERK, and RSK, all core kinases activated during growth signaling, downstream of receptor tyrosine kinases (RTKs) and phosphatidyl inositol 3-kinase (PI3K). PTEN turns off PI3K-derived signaling events by reducing PI3P levels. mTORC1 is also regulated by the rag GTPases which are inactivated by the GATOR1 complex, made up of DEPDC5, NPRL2, and NPRL3. mTORC1 stimulates cell size increase and growth through several anabolic effects (not shown). Inactivating mutations in any of TSC1, TSC2, DEPDC5, NPRL2, or NPRL3 can lead to mTORC1 activation. Activating mutations in either RHEB or MTOR can lead to mTORC1 activation. The mTOR protein occurs in either of two large protein complexes, mTORC1 and mTORC2. Arrows indicate stimulatory events, which at times are mediated by phosphorylation (+ P); blocked lines indicate inhibitory effects.
